# The Relation of the Chemistry and Bacteriology of the Stomach to Dental Caries

**Published:** 1897-10

**Authors:** J. H. Kellogg

**Affiliations:** Battle Creek, Mich.


### THE DENTAL REGISTER.

VOL. LI.] OCTOBER, 1897. [No. 10.

### COMMUNICATIONS.

### The Relation of the Chemistry and Bacteriology of the Stomach to Dental Caries.

BY J. H. KELLOGG, M. D., BATTLE CREEK, MICH.

That a relation exists between disorders of the stomach and premature decay of the teeth has long been recognized as an accepted clinical fact, but exactly what this relation is, I am not aware that any one has heretofore undertaken to define. In presenting this paper, devoted to the consideration of this subject, I do not assume to treat the subject exhaustively, nor to say the last word upon the subject. My paper is based simply upon such observations as I have incidentally made in connection with the study of the conditions of the stomach by exact methods of chemical and bacteriological research. My paper comprises the results of the study of the teeth in one hundred cases of persons suffering from various forms of disordered digestion.

First, a word with reference to the methods of research employed:

*The Method of Chemical Examination.*—This consists in a determination of the constituents of the stomach fluid obtained after a test-meal consisting of one and one-half ounces of water-free, unleavened bread or granose, a preparation of wheat, with seven and one-half grains of common salt, or chloride of sodium, and eight ounces of water. The methods of examinatiom employed are the same as those used in the assaying of ores, or the quantitative analyses of compounds of any sort in well-equipped chemical laboratories. The general plan of chemical investigation is that which has been developed in the Laboratory of Hygiene of the Battle Creek Sanitarium within the last four years, consisting of the excellent methods of Hayem and Winter, the method of Toepfer, and other methods which have been the outgrowth of necessities developed during the progress of the work.

The bacteriological research conducted simultaneously with the chemical examination employs the latest methods of bacteriological research for both ærobic and anærobic micro-organisms, and a biological study of the character of the special ptomains or toxins developed in the stomach as the result of bacterial growth. By the aid of these methods the following particulars in relation to a given stomach fluid are obtainable:

1. The total acidity, representing the combined acidity, consisting of free hydrochloric acid, loosely-combined chlorin, or acid albumin, and various organic acids, as lactic, acetic, butyric, etc.

2. The total amount of chlorin separated. This furnishes a basis for classification in relation to the quantity of chemical work done by the stomach with reference to its secretory or granular activity.

3. The amount of free HCl.

4. The amount of organically-combined chlorin.

5. The amount of acid combined chlorin, representing the amount of chlorin which has actually entered into the digestive process in the formation of normal products.

6. The amount of neutral chloro-organic compounds, products due to morbid chemism or to the bacteriological fermentation of proteids.

7. The amount of organic fatty acids, such as lactic, butyric, and formic acid, the result of the fermentation of farinaceous saccharin, and other food elements in the stomach.

8. The amount of maltose, or digested starch, and of soluble starch and dextrin or partially-converted starch.

9. The presence in proper quantity, and the deficiency or absence of pepsins and other ferments or enzymes.

10. The presence in normal quantity, and deficiency or absence of starch-digesting ferments in the saliva.

11. The number of bacteria per cubic centimeter of stomach fluid. The normal stomach fluid contains no bacteria at the end of the first hour of digestion after a sterile test-meal of bread and water.

12. The character of the micro-organisms present, whether yeast, mould, or bacteria.

13. The character of the dominant micro-organisms, whether acid-forming, gas-forming, gelatine-liquefying, or milk-coagulating, and whether pathogenic or non-pathogenic.

14. The character of the products of the micro-organisms present in the stomach fluid when grown in different media, and whether toxic or not toxic.

15. The presence or absence of mucus, blood, pus, and other morbid elements in the stomach fluid.

16. The position of the stomach and other digestive organs, whether normal or prolapsed, and to what extent.

The exact mathematical data thus obtained render possible the determination of a series of highly-important co-efficients, whereby the physician is able to judge at a glance of the percentage degree to which any phase of the digestive process may be in divergence from the normal condition. Each of the following co-efficients has been worked out by the author, excepting one co-efficient, *a*, which has been modified from a similar co-efficient first determined by Hayem and Winter:

1. Proteid digestion (*a*). This co-efficient shows the relation existing between the acid combined chlorin and the total combined chlorin; a low percentage indicates a small amount of normal digestive activity, with the formation of a large amount of non-usable and toxic chloro-organic compounds, the result of a morbid chemism and the bacterial fermentation of proteids, a condition found present in the majority of cases of nervous dyspepsia, migraine, and other forms of chronic toxemia.

2. Starch digestion (*b*)—(1). The relation of the amount of perfectly-digested starch or maltose to the dextrin and soluble starch, or imperfectly-digested starch; (2) the relation of the amount of maltose or completely-digested starch to the normal amount.

3. Salivary activity (*c*). The degree of salivary activity, as shown by a single expression representing both the quantity of the saliva formed in a given time and the digestive activity of the same.

4. The relation of the amount of HCl separated to the total chlorine (fixed, free and combined) found in the stomach fluid (*m*).

5. The relation of HCl separated to the amount normally separated (*n*).

6. Fermentation (*x*), as shown by the number of bacteria and the quantity of fatty acids and other products of fermentation present.

7. Solution (*y*), as shown by comparison of the undigested residue with the total amount of stomach fluid.

8. Motility (*z*), based upon the careful measurement of the residual fluid contained in the stomach at the end of the first hour of digestion. This is measured by an exact method described in another paper.

9. Capacity (*k*), determined by the comparison of the height of the individual in millimeters with the capacity of the stomach in cubic centimeters. The normal stomach has one cubic centimeter of capacity for each millimeter of the individual's height.

By means of such an examination as this it is possible to make a complete and exact classification of stomach fluids or cases. The study of the results of more than eight thousand examinations, relating to over six thousand individual cases, has resulted in the reference of all classes of functional derangement of the chemical processes of the stomach to one of four classes, as follows:

Hyperpepsia, in which there is an excessive separation of HCl.

Hypopepsia, in which there is a deficient separation of HCl.

Apepsia, in which there is a total absence of digestive work.

Simple dyspepsia, in which the quantity of work done is normal, the failure being in quality only.

In the study of the teeth in a hundred cases in which this analysis and classification has been made, I have ascertained, in addition to the name of the patient, the age, the condition of the teeth, the number of sound teeth, the number of filled teeth, at what age the teeth began to decay, whether or not symptoms of indigestion were noticed either before or about the same time the decay of the teeth began, whether any connection between the decay of the teeth and the condition of the stomach has been observed, and whether soft foods have been largely used instead of foods requiring mastication.

In making a study of the relation of dental decay to the various classes of indigestion as shown by chemical analysis of the stomach fluid, I noted the following facts:

1. Of the one hundred cases, two only had perfectly sound teeth; one a case of hyperpepsia, the other a case of simple dyspepsia. The fact that in one hundred cases of indigestion but two persons were found with sound teeth, and these cases in which the digestive disturbance was comparatively slight indicates at the outset, that there must certainly be a very important relation between dental decay and gastric disorder. But we have, in this fact alone, no intimation as to the character of the existing relationship.

2. The average number of sound teeth found in fifty-one cases of hypopepsia was ten; in forty cases of hyperpepsia, fourteen; in nine cases of simple dyspepsia, twenty. The fact that the number of sound teeth found in these three great classes of cases was, in hypopepsia ten, in hyperpepsia fourteen, and in simple dyspepsia twenty, indicates a progressive deterioration in the teeth from simple dyspepsia through hyperpepsia to hypopepsia. This observation agrees with the observed facts in relation to the clinical history of functional disorders of digestion in which the stomach chemism is disturbed. All classes of cases doubtless begin, in the most cases at least, with simple dyspepsia. As the disease advances, hyperpepsia appears, and later we find hypopepsia in the completely worn-out stomach.

From these facts we must conclude, either that the teeth deteriorate with the stomach, as the result of the action of some cause operative upon both the teeth and the stomach, from constitutional deterioration; or that the progressive deterioration of the stomach reacts injuriously upon the teeth. The additional data which I will give may throw some further light upon this point.

An inquiry respecting the extent to which the teeth were entirely destroyed in the different classes of indigestion showed that in nine cases of simple dyspepsia no person has been obliged to resort to the use of artificial teeth. In the forty cases of hyperpepsia, three persons (or seven and five-tenths percent) were wearing artificial teeth, two, single sets of teeth (upper), and one, both upper and lower sets. In hypopepsia twelve persons (twenty-three and five-tenths percent) were provided with artificial teeth, five with upper sets only, and seven with both upper and lower sets. The proportion of persons who had completely lost one set of teeth was found to be: for simple dyspepsia, zero; for hyperpepsia, five percent, and for hypopepsia, ten percent. And the percentage of those who had lost both upper and lower teeth so completely as to require artificial teeth, was: for simple dyspepsia, zero; hyperpepsia, two and one-half percent; for hypopepsia, fourteen percent, or nearly six times the rate for hyperpepsia. These facts show conclusively that the most advanced of dental decay is found associated with hypopepsia.

3. In a study of the condition of the teeth in relation to gastric catarrh, as shown by an excessive quantity of mucus present in the stomach fluid, I noted the following: In thirty cases of hypopepsia with gastric catarrh, the average number of good teeth present was twelve, and in twenty-one cases of hopopepsia without gastric catarrh, the number of good teeth was nineteen, or more than fifty percent more teeth in cases in which gastric catarrh is not present. In eleven cases of hyperpepsia with gastric catarrh, the average number of good teeth was found to be twelve. In twenty-nine cases of hyperpepsia without gastric catarrh, the number of good teeth was twenty, or sixty-six and two-thirds percent more teeth in cases of hyperpepsia without gastric catarrh than in cases of hyperpepsia with gastric catarrh. In five cases of simple dyspepsia with gastric catarrh, the average number of good teeth was found to be nineteen; in cases of simple dyspepsia without gastric catarrh, the average number of sound teeth was twenty-four.

4. A study of the relation of free hydrochloric acid in the gastric juice to dental decay gives the following results: In cases of hypopepsia in which more than ten milligrams of free HCl was found in the stomach fluid, the average number of good teeth was twenty-three; in hyperpepsia, sixteen; in simple dyspepsia, twenty. It is very interesting to note that in cases of hypopepsia in which hydrochloric acid was present to the amount of ten milligrams per 100 c. c. or more of stomach fluid, the average number of sound teeth found in the whole number of cases of hypopepsia was only ten. In cases of hyperpepsia, with twenty-five or more milligrams of free HCl per 100 c. c., the average number of good teeth was found to be nineteen, while the average in the total number of cases was but fourteen. In simple dyspepsia the average number of sound teeth in cases with twenty-five or more milligrams of free HCl per 100 c. c. of stomach fluid was twenty-four, whereas the average in simple dyspepsia was twenty.

These facts show clearly that the presence of free hydrochloric acid in the stomach fluid is a decided advantage to the teeth, the average number of sound teeth when free hydrochloric acid is present in a reasonable amount being 230 percent greater in hypopepsia, thirty percent greater in hyperpepsia, and twenty percent greater in simple dyspepsia. It would, of course, be expected that the influence of free hydrochloric acid would be relatively much greater in hypopepsia than in hyperpepsia or simple dyspepsia, for the reason that the absence of free hydrochloric acid is one of the most characteristic features in hypopepsia, whereas HCl is usually present in sufficient quantity in simple dyspepsia and hyperpepsia.

5. In eighteen of the one hundred cases microbes were found present in the stomach fluid to the amount of 25,000 or more for each cubic centimeter of the stomach fluid. The average number of sound teeth found in these cases was eleven and one-third. In eight cases, or forty-four percent of the entire number, there were no sound teeth; and in six, or thirty-three and one-third percent, the natural teeth had been entirely replaced by artificial teeth. I call attention, in passing, to the fact that of the eight cases in which the teeth were entirely lost, six, or seventy-five percent, had been replaced, and in eighteen cases microbes were present to the number of 25,000 or more per cubic centimeter of stomach fluid, making the proportion of cases more than four times greater for this class than for the total number.

A study of the special characteristics of the germs found in these cases showed both acid-forming and gelatine-liquefying germs to be present in all but two cases; and in those two cases acid-forming germs did not grow in the culture media, but the gelatine-liquefying germs were present, and the stomach fluid had an exceedingly offensive odor in both. All the teeth had been lost in both these cases.

These facts show most conclusively an important relationship existing between an infected condition of the stomach and dental caries, a confirmation of the observation that the number of good teeth found present increases with the amount of free hydrochloric acid present in the gastric juice. The facts gleaned do not indicate anything in relation to the character of the germs, or their specific characteristics. Both acid-forming and gelatine-liquefying germs were present in nearly every case; both classes of germs were probably present in all cases, the absence of acid-forming germs in the two cases referred to being probably accidental, or due to the fact that the growth of the acid-forming microbes present was checked by the presence of the other microbes inimical to them. It thus appears that microbes capable of producing both acid or proteid or putrefactive fermentations were present in the stomach fluid in all cases exhibiting the highest degree of dental decay. The products of acid fermentation are capable of attacking calcareous structures, and recent observations have shown that the bacterial agents of putrefaction produce digestive ferments capable of attacking and dissolving proteid or albuminoid substances. It will thus be readily seen that the two classes of germs present are well adapted to the destruction of the peculiar histological structures which form the teeth.

The question naturally arises whether it is proper to draw the conclusion that the presence of germs of any particular class in the stomach is evidence of the presence of the same germs in the mouth. Upon this point I think it safe to affirm that no germs are ever found in the stomach which have not previously passed through the mouth, even if they did not remain long enough to be considered as regular inhabitants of the oral cavity. Repeated examinations of the fecal discharge of the new-born infants have shown that the alimentary canal of human beings when born into the world is absolutely free from microbes. Pasteur, many years ago, ventured the conjecture that microbes are necessary to the accomplishment of the digestive process through the action of the several digestive ferments which are produced by them. Tieghem subsequently undertook to demonstrate that the bacillus amylobacter performs an important function in digestion by the transformation of the cellulose of plants in assimilable substances. But a series of experiments carried out under my direction in the Laboratory of Hygiene of the Battle Creek Sanitarium, during the last two years, has demonstrated that in the majority of healthy persons the stomach contents are found perfectly sterile at the end of the first hour of digestion after the administration of a sterile test-meal of cooked cereals and water.

In cases of hyperpepsia also, the stomach fluid is usually found sterile, and not infrequently no living microbes or spores are to be found after a sterile test-meal, even in cases of simple dyspepsia and hypopepsia. The stomach evidently possesses the ability to destroy both microbes and their spores, not only through the presence of the hydrochloric acid which it secretes, but by some other means similar to that by which other mucous surfaces, such as that of the nose, are able to maintain a sterile condition.

The ability of the living body to defend itself against microbes is a facility which it apparently possesses in common with all higher organisms and by healthy plants as well as animals, it now being well known that plants produce upon the surface of their bark and leaves a substance allied to, if not identical with, formalin, whereby microbic life is destroyed and prevented from establishing parasitic colonies.

Since my own observations respecting the ability of the stomach completely to sterilize itself, Nuttall and Thierfelder have demonstrated that microbes are not essential to animal life, by placing a guinea-pig born by Cæsarian section under aseptic conditions in a specially-constructed apparatus in which it could be carefully supplied with food, air and water without the admission of microbes. At the end of eight days the animal had consumed thirty-five grams of food and gained ten grams in weight; it had actually increased in weight more rapidly than some of its brothers born at the same time who had been nourished under ordinary conditions. The animal was then killed, carefully dissected, and examined bacteriologically, but not a single microbe was found any where in its alimentary canal or in any other portion of its body.

Kurhoff and Wagner also recently published in the *Central. blatt fur Bakteriologie und Parasitenkunde*, the results of an investigation conducted by them which show conclusively that there are no microbes which are habitual parasites of the stomach, the stomach being capable of destroying all bacteria, except those which contain spores, within half an hour after they are received into its cavity.

A study of the microbes of the mouth by Miller, Vignal, Biondi, Pasteur, Klein, Sternberg, Frænkel, Netter, Corneil, Weichselbaum, and others, has developed the fact that under ordinary conditions a large number of species of microbes are to be found resident in the mouth.

Biondi isolated five species, all of which were pathogenic and capable of producing septic conditions and suppuration. Vignal, in 1887, described nineteen species of micro-organisms which he had isolated from the mouth; seven of these were capable of dissolving cooked albumin; ten dissolved fibrin; nine dissolved gluten; three possessed diastatic properties, converting starch into sugar; seven coagulated milk; six dissolved casein; seven produced lactic acid; seven were capable of inverting cane-sugar, and seven set up alcoholic fermentations in solutions of glucose.

Six of these micro-organisms were found capable of resisting the action of the gastric juice to a considerable extent; fourteen showed ability to resist the action of gastric juice for some time; the remaining five were killed within half an hour after exposure to the gastric juice.

Vignal added to these observations another important fact, demonstrating the relationship between the organisms of the mouth and those of the alimentary canal, by identifying in the fecal matters six of the organisms which he had found in the mouth. Pasteur isolated more than thirty different microbes from fluids withdrawn from the stomach. Abelous isolated sixteen different species from the fluids obtained from his own stomach by washing his stomach when empty. Gillespie isolated twenty-four species of bacteria from his own stomach.

In over 2,000 bacteriological examinations of stomach fluid which have been made in the Laboratory of Hygiene of the Battle Creek Sanitarium, a very large number of different species of bacteria have been observed; but as the work of isolating and identifying these is not yet completed, I am not able to speak more definitely than I have already done concerning the number of different species of microbes to be found in the stomach. This point, however, is not essential to this paper, my sole purpose being to show that the microbes found in the stomach are, in a general way, identical as regards number and characteristics, with those found in the mouth.

A clinical evidence of a relationship between the microbes of the mouth and those of the stomach is to be found in the prolific bacterial growth commonly found upon the tongue and teeth in cases of chronic indigestion. From the most ancient times the condition of the tongue has been recognized as an important indicator with respect to the functional state of the stomach. That these organisms are by no means modern is evidenced by the recent demonstration of *leptothrix buccalis* upon the teeth of a mummy by Sopf and Miller in their study of prehistoric teeth.

In one hundred cases, upon the study of which this paper is based, a brown, slimy, or otherwise infected tongue was found in every case. That a large number of germs was found in the stomach fluid in only eighteen percent of the entire number was due to the fact that the stomach had not yet lost its power to sterilize its contents.

As regards the question as to which are first in order, the microbes of the mouth or those of the stomach, it must be granted that infection of the stomach must come through and by the mouth, and yet the facts which have been brought forward in this paper clearly show that the condition of the mouth in some way depends upon the state of the stomach as well as the reverse. Clinical experience also agrees with this, since, as is well known, a disturbed state of the stomach, as a bilious condition, will quickly give rise to a thick coat upon the tongue, which is nothing more nor less than a prolific bacterial growth.

It must be granted that the mouth is, under ordinary conditions, as well prepared to defend itself against the attacks of microbes as is the nose, the stomach, and other mucous surfaces. The rapid development of bacterial growths in the mouth when the stomach is disturbed must be due to some relation existing between the two whereby an infected condition of the stomach destroys the power of the mouth successfully to maintain its resistance against micro-organisms. With reference to the exact character of this relationship we are not perhaps prepared to speak with positiveness; but I will venture to suggest as a hypothesis that when the stomach loses its power to maintain a sterile condition, the microbes which are constantly entering it from the mouth and nose, and in the greatest abundance perhaps in the food, develop so rapidly as to get the ascendancy, and produce in large quantities ptomains and other toxic substances which, when absorbed into the circulation and distributed throughout the body infect the whole organism, and lessen its ability to maintain active resistance against the encroachments of bacterial parasites.

This hypothesis is in line with the fact that typhoid fever, chlora, and other infectious disorders whose seat of attack is in the alimentary canal, are powerless to obtain a foothold in the body unless the way has been prepared for them by previous functional disorders of the stomach whereby its resistance is diminished. If this hypothesis is correct, the mouth suffers in common with the rest of the body, and the thickly-coated condition of the tongue is chiefly the result of the fact that the mouth is, more than other portions of the body, exposed to the attacks of organisms through furnishing them a convenient harbor, and at the same time providing nutrient material in the form of fragments of food left between the teeth as the result of imperfect cleansing.

This theory seems plausible in view of the readiness with which disorders of other mucous surfaces, such as nasal catarrh, obtain a foothold during a temporary attack of indigestion in the case of persons who at other times are not subject to such disorders. At the same time it must be readily seen that the infected condition of the mouth must lead to and aggravate a disordered condition of the stomach. It may be very reasonably suspected that catarrh of the stomach is due to infection from the nose and mouth after the stomach has lost its power to destroy the organisms introduced into it. An infected mouth must lead to infection of the food, and, through it, to infection of the stomach, in case the stomach has to a sufficient degree lost its ability to defend itself against the invasion of parasitic organisms; hence, we may readily see how neglect to cleanse the mouth properly may lead to disorder of digestion and even to such microbic maladies as intestinal catarrh, infectious jaundice, and a variety of intestinal disorders.

In reply to the question, "Has any connection between decay of the teeth and the condition of the stomach been observed," I would answer, in thirty-two percent of the cases examined and reported, decay of the teeth was noted before or in connection with indigestion. In eighteen percent, decay was observed after the first symptoms of indigestion were noted. In fifty percent, no observation was made.

These replies accord with the facts previously noted, and warrant us in suggesting that in the intimate relation which evidently exists between decaying teeth and functional disorders of the stomach, the condition of the stomach is the primary fault, although a disordered condition of the teeth evidently aggravates and intensifies stomach disorders.

I have made many further observations in regard to the influence of diet upon the teeth, etc., but these may be more appropriately reported in another paper.

It is not within the scope of this paper to consider in detail the important subject of the hygiene of the teeth, but it may be proper in this connection to add a word or two upon this subject:

1. It is evident that if the health of the teeth depends to any considerable extent upon the condition of the stomach, any thing which interferes with the digestive functions will to some extent militate against the health of the teeth. That dental caries and the various septic and inflammatory processes to which the teeth and contiguous structures are subject are directly due to the action of microbes is a question which is no longer debated. Hence, it is self-evident that anything which encourages the development in the mouth of the microbes which are parasitic in this region of the body must encourage dental decay.

An inquiry respecting the character of the food, as to its consistency in the case of each one of the one hundred persons to whom this paper relates, developed the fact that the average number of good teeth in persons using soft food was, for hyperpepsia, fourteen; for hypopepsia, eighteen; for simple dyspepsia, nineteen; whereas, the average number of good teeth in persons using habitually a portion of hard food was, for hypopepsia, sixteen; for hyperpepsia, nineteen; and for simple dyspepsia, twenty-three. The average number of sound teeth in all persons using soft food exclusively was but eleven; whereas, the average number of good teeth in persons using hard food was twenty-four and one-half; in cases of those using mixed food, nineteen.

Some very interesting experiments conducted in the Laboratory of Hygiene of the Battle Creek Sanitarium a couple of years ago have led me to the discovery of the fact that the principal virtue in hard food is not its hardness but its dryness. The leading facts upon which this conclusion is based I quote as follows from a paper entitled, "Experimental Researches Relating to Salivary Secretion and Digestion:"

"The quantity of food or fluid was, in each case, one ounce.

The food, whether solid or fluid, was taken into the mouth in small portions and chewed for a few seconds, then being rejected from the mouth, it was received into a small vessel for weighing. The length of the experiment with each substance lasted exactly five minutes. The difference in weight between the substance after chewing and before, represented the amount of saliva which had been added to it during mastication.

"One ounce of granose increased in weight 59.79 grams.

"One ounce of granose with two grams of common salt increased in weight 58.80 grams.

"One ounce of granose sprinkled with pepper increased in weight 59.1 grams.

"One ounce of granose with 5 c. c. of strong cider vinegar increased in weight 55.9 grams.

"One ounce of moist bread increased in weight 31.1 grams.

"One ounce of raw apples increased in weight 38.1 grams.

"One ounce of water increased in weight 2.92 grams.

"One ounce of milk increased in weight 3.82 grams.

"One ounce of pea soup increased in weight 5.82 grams.

"Paraffin chewed for five minutes produced 20 c.c. of saliva."

The above facts show very clearly the importance of the quality of dryness in relation to gastric and dental hygiene. There are many persons whose teeth are already so far damaged that the use of hard foods, such as zweiback and very hard crackers, is practically impossible, and the attempt to use such articles of food results in the introduction into the stomach of large, hard masses of imperfectly-masticated food, which, being retained for a long time in the stomach, give rise to indigestion, so that the attempt to use hard foods aggravates the difficulty instead of diminishing it.

In attempting to meet the wants of this class of cases I have been led to prepare a cereal product consisting of whole wheat grains compressed into the form of very thin flakes which have the quality of crispness and dryness, and stimulate the salivary glands to a high degree while at the same time taxing the dental structures only to a very limited extent. I have found this food of very great service in the treatment of patients whose teeth are so far destroyed as to make the use of hard food impossible.

It is easy to understand that the employment of soft food leads to deterioration of the teeth by the development of disorder of the digestive process. Soft food is swallowed without proper mastication. The omission of this link in the chain of the digestive process necessarily leads to gastric derangement and to derangement of each successive step in the series of changes whereby the food is elaborated in the blood and tissues. This being true, it is also true that all articles of food and all dietetic practices which disturb the digestive processes must encourage dental decay.

Experiments of Sir William Roberts and others have shown that tea, coffee, beer, wine, and vinegar are particularly productive of indigestion through interference with the action of the normal ferments which act upon the food, particularly the diastatic ferment of the saliva. Dr. Roberts showed that these substances, when present even in a very small quantity, prevent the action of the saliva in transforming starch into sugar. Hence, these substances must all be indicated in the cases of persons whose teeth show a tendency to decay, and it scarcely need be added that it would be well to encourage all people who wish to preserve their teeth intact to a good old age to give articles of the soft material a wide berth.

Cheese and other foods in which putrefactive processes and living microbes innumerable are always present ought to be totally discarded as fit only for scavengers, and not suited to the delicate digestive and comparatively-feeble disinfectant or sterilizing capacity of the human stomach.

I think it must also be conceded that in cases of hypopepsia, in which there is a decided deficiency of free HCl and hence inability on the part of the stomach to sterilize its contents perfectly, the question may be raised whether some substitute may not be wisely sought for, for flesh meats which so readily undergo putrefactive processes in the alimentary canal, particularly red meats, such as beefsteak, roast beef and mutton chops, fish and shell fish, all of which leading French physicians, following the teachings of the late eminent Dujardin-Beaumetz, invariably interdict in hypopepsia and in cases of diminished gastric motility, gastric catarrh, and dilatation of the stomach.

It has also been suggested, and I think very justly, that the use of flesh meats encourages dental caries by the retention of bits of muscular fiber between the teeth, furnishing an admirable culture media for those germs which are most destructive of the teeth. This objection may of course be largely removed by a proper cleansing of the teeth, but unfortunately it is quite probable that some time must yet elapse before the average man becomes sufficiently civilized to appreciate the necessity for washing his mouth with the same regularity as he would wash his face and hands.

I confess I am somewhat sceptical as to the primitive necessity for this habitual mouth-cleansing; I think it must be said that tooth-washing is a thing as peculiar to the human race as is smoking and the wearing of clothes, both of which practices are entirely artificial, and I venture the suggestion that tooth-washing is an artificial rather than a natural necessity, and that it is the result of erroneous habits in diet which give rise to artificial and injurious conditions pertaining to the teeth. It is a noteworthy fact that dental decay is by no means so common among savages as among civilized people, except in cases of those savages who have adopted the eating habits of civilization. The regular, white, sound teeth of the Southern negro who subsists upon a simple dietary of corn bread, boiled beans, and a taste of bacon now and then, he doubtless owes to the fact that he is not yet sufficiently civilized to be deprived of the use of his instruments of mastication.

Dental caries, as is well known, is exceedingly rare among lower animals when fed upon a natural dietary, but among civilized people everywhere, particularly among the wealthier classes, dental caries is advancing at a rapid rate in spite of the vigorous use of the tooth-brush, and the assiduous attention of thousands of dentists, whose prosperous associations, well-equipped offices, fine turn-outs, and magnificent dwellings are abundant evidence of the prosperous state of their profession. There is certainly room for the query whether there is not a most excellent prospect that the human race will not at some future time become wholly toothless, and as dependent upon artificial means for the mastication of food as we now are upon fire and culinary utensils for cooking it. A wag has suggested that the gold mines of some future generation will be found in the cemeteries of the present.

This question is certainly one which ought to receive most careful and assiduous attention, and I believe it would be to the interests of humanity, if not to the financial interest of the members of this association, if you would empower a committee to prepare a series of questions for research and study through observation and experimentation, whereby further light might be thrown upon the question which has formed the subject of this paper. I should be very glad to co-operate with such a committee in a research of this kind.

The facts that I have undertaken to present in this paper may be briefly summarized as follows:

1. Dental caries is almost universal in cases of chronic indigestion; only two persons in one hundred were found to have sound teeth.

2. The largest number of sound teeth were found in simple dyspepsia; the smallest number in hypopepsia.

3. In hypopepsia with gastric catarrh the number of sound teeth averaged but twelve; whereas, in simple dyspepsia without gastric catarrh the average number was twenty-four.

4. The highest degree of dental caries was found in cases in which the amount of free hydrochloric acid present was less than ten milligrams per hundred c. c. of stomach fluid.

5. The average number of sound teeth in persons whose stomach fluid contained 25,000 or more microbes per cubic centimeter was but eleven and one-third.

Complete loss of teeth was found to be four times as frequent in this class of patients as in dyspeptics as a class, showing that bacterial infection of the stomach is closely associated with dental caries.

6. A study of the physiological characters of the germs present in the stomach in these cases showed them to be both acid-forming and gelatine-liquefying, that is, capable of producing both acid fermentation and putrefaction.

7. Bacterial infection of the mouth is an abnormal condition, and may lead to infection of the stomach and alimentary canal in general. Bacterial infection of the stomach leads to infection of the mouth.

8. The study of the one hundred cases considered in this paper shows that the beginning of decay of the teeth is as a rule either co-incident with gastric indigestion or subsequent to it, hence it is evident that the hygiene of the mouth and the teeth necessarily includes the hygiene of the stomach and careful attention to dietetics, and will lead to the exclusion of all substances which impair the digestive functions, and especially those substances which encourage bacterial infection of the mouth and stomach.

9. The employment of soft food encourages deterioration of the teeth, through the development of disorders of digestion.

### DISCUSSION.

#### AFTERNOON SESSION—WEDNESDAY.

Discussion of Dr. Kellogg's paper: "The Chemistry and Bacteriology of the Stomach in Relation to Dental Caries."

Dr. Hoff: Dr. Kellogg has suggested to the dental profession a work that, with his help, may be pursued to profit. I don't know that I understood him to convey the idea that decay necessarily follows these indications of dyspepsia or indigestion, or that this was his inference. It has been our habit always to look at this subject from the other direction. We have always thought that disease of the digestive functions followed diseases of the teeth. We have looked at it from another standpoint to-day through Dr. Kellogg which, it seems to me, is worth some very serious consideration and further investigation. I think that nothing better could be done than the appointment of a committee by the president, as the doctor has suggested, to assist him or work under his direction in this matter. My idea of what this paper was to be, was that it was to give us some idea of what influence these diseases have upon diseases of the mouth, other than dental caries. We have considered diseases of the teeth more as a local affection. There are numerous diseases of the gums and mucous membrane of the mouth of systemic origin, a consideration of which would not be pertinent to the subject which Dr. Kellogg has presented. I feel entirely incapable of intelligently or profitably discussing the subject as presented, because it is new to me and comes a pleasant surprise, that I had rather think than talk about. I am sure all of us have questions we would like to ask Dr. Kellogg, and will give way for this purpose.

Dr. Kellogg: I do not feel that I am competent to preside over a question box. I am obliged to say that the dental profession has got ahead of the medical. The doctors all know that when they meet a body of dentists, they meet a body of scientific and progressive men. I will say that the one hundred patients referred to ranged from twenty to sixty years. The average age was about forty. I have noted down the time at which decay began. I have found that in a large percentage decay occured after indigestion began. I do not wish to be understood to say that all the causes of decay of the teeth come from the stomach. There are conditions both ways. Most of these disorders of the mouth are said to come from bacteria. They are certainly bacterial in their origin. Germs of all sorts attack the body because it has lost its power to defend itself.

Dr. Parker: I have observed, as a dentist, that the most active period of decay was between the ages of fourteen and twenty.

Dr. Siddall: I know two men sixty years of age who have perfect sets of teeth. Those are all that I know.

Dr. Loeffler: There is an article in Dr. Miller's book in regard to the action of bacteria in the stomach, and one question I would like to ask: He has made experiments on the action of bacteria in normal stomachs; experiments upon dogs for instance, to show that bacteria taken up in the mouth will entirely pass through the stomach and alimentary canal, provided they are taken at the first of the meal. I understood Dr. Kellogg to say that bacteria, no matter of what nature, were destroyed.

Dr. Kellogg: I believe the stomach is capable of destroying all kinds of germs.

Dr. Loeffler: I think in these experiments, on examination of the food after it had left the alimentary canal, germs were found that were introduced with the food in these experiments performed on dogs. That seemed to be in opposition to what was said by the doctor. I simply made this statement as I read it, though the condition of the stomach of a dog may be different from that of a person.

Dr. Kellogg: I would simply say that it was a difference in the character of the meal. It is necessary that it should be a sterilized meal. In a test meal of meat or beef tea, you will find any number of germs in the healthy stomach. When of sterilized cereals, granose or zwiebach, you will find no germs at all. I will mention how this discovery was made. I invited Professor Novy to come to Battle Creeek, that we might work together on a certain experiment. We began our investigations on healthy persons. I furnished him a number of healthy persons and they had a test meal. After he had been at work a week he said, "Doctor, I don't know what is the trouble, but I can't find any germs." I asked him if he was sure his methods were all right. He said he was. We made a catalogue of the germs present in the normal stomach. He found no germs in several weeks more. I changed the test meal, but it was purely accidental. Made the meal largely of water biscuit. The germs began to appear in large numbers. In some cases no germs, but in many, large numbers of them. These germs were traced back to the flour. The baking of the biscuit was not sufficient to destroy them. I presume that it was probably true with Dr. Miller, that he did not have a sterilized meal. The investigations by Wagner and Herhoff have shown that the stomach will destroy all ordinary germs in a half hour, and every kind of germ will be destroyed in the stomach if held there a proper length of time. If we introduce a large mass of germs, they may be forced out before disinfection takes place. If they get to the intestines, they are sure to grow in numbers. We have found hundreds of persons whose stomachs were absolutely sterile after a test meal. Since the investigation of Herhoff and Wagner, which was reported here a few weeks ago, I have felt encouraged to believe that the investigations in our labratory would stand the test of further investigation.

